# A spatial model to predict the incidence of neural tube defects

**DOI:** 10.1186/1471-2458-12-951

**Published:** 2012-11-07

**Authors:** Lianfa Li, Jinfeng Wang, Jun Wu

**Affiliations:** 1 State Key Lab of Resources and Environmental Information Systems, Institute of Geographical Sciences and Natural Resources Research, Chinese Academy of Sciences, 1305, No. A11, Rd. Datun, Anwai, Beijing, 100101, China; 2 Program in Public Health, College of Health Sciences, University of California, Irvine, USA; 3 Department OF Epidemiology, School of Medicine, University of California, Irvine, USA

**Keywords:** NTD, Birth defects, Residual, Spatial model, GAM

## Abstract

**Background:**

Environmental exposure may play an important role in the incidences of neural tube defects (NTD) of birth defects. Their influence on NTD may likely be non-linear; few studies have considered spatial autocorrelation of residuals in the estimation of NTD risk. We aimed to develop a spatial model based on generalized additive model (GAM) plus cokriging to examine and model the expected incidences of NTD and make the inference of the incidence risk.

**Methods:**

We developed a spatial model to predict the expected incidences of NTD at village level in Heshun County, Shanxi Province, China, a region with high NTD cases. GAM was used to establish linear and non-linear relationships between local covariates and the expected NTD incidences. We examined the following village-level covariates in the model: projected coordinates, soil types, lithodological classes, distance to watershed, rivers, faults and major roads, annual average fertilizer uses, fruit and vegetable production, gross domestic product, and the number of doctors. The residuals from GAM were assumed to be spatially auto-correlative and cokriged with regional residuals to improve the prediction. Our approach was compared with three other models, universal kriging, generalized linear regression and GAM. Cross validation was conducted for validation.

**Results:**

Our model predicted the expected incidences of NTD well, with a good CV R^2^ of 0.80. Important predictive factors included the fertilizer uses, locations of the centroid of each village, the shortest distance to rivers and faults and lithological classes with significant spatial autocorrelation of residuals. Our model out-performed the other three methods by 16% or more in term of R^2^.

**Conclusions:**

The variance explained by our model was approximately 80%. This modeling approach is useful for NTD epidemiological studies and intervention planning.

## Background

Birth defects refer to functional or structural anomaly present in infancy or later in life that can result in infant mortality and disability. Neural tube defect (NTD), one of the most common types of birth defects, have been estimated to occur in more than 320,000 infants worldwide annually [1]. NTD have a multifactorial etiology, with both genetic and environmental contributions [2]. Known risk factors for NTD include folate deficiency, maternal epilepsy with concomitant anticonvulsant drug exposure, maternal obesity, diabetes mellitus, and maternal hyperthermia [3].

Environmental pollutants have also been linked to NTD [3-6]. Chisholm et al. [7] and Bai [8] associated high levels of disinfection by-products with the prevalence of NTD and other birth defects using logistic regression. Spatial distributions of soil and rock types were linked to the incidences of NTD in [9] and [10]. Zhang [11] and Li [12] found a marginal correlation (p-value < 0.1) between the association of NTD incidence and chemical elements in soils (e.g. Mn and Cr). Certain soil types may be associated with lower levels of important nutritional elements [11-13] that in turn have influence on fetal growth. Recently, the spatial distribution of NTD has been modeled for its temporal trend [10], regional correlation [14], and association with distance to rural coal mining area [15]. Wang et al. [16,17] used support vector machine to predict the risk of NTD at three levels (none, low to medium and high). Wang et al. [9] also quantified the risk of NTD using a geographical detector and three types of environmental factors (primary physical variables such as watershed, lithozone and soil; basic nutrition such as food; ancient materials from geological faults).

Although spatial analysis was used to examine the effects of the environmental factors on birth defects, it was not used to directly predict the incidence of NTD in Heshun, an area with extremely high rate of NTD. In addition, it is seldom used to quantify potentially important features (e.g. soil and lithodological types, faults and rivers) for combinational use with other numeric influential factors (e.g. use of pesticides and disinfection products, population, income and number of doctors) [9]. Further, the spatial clustering or autocorrelation was observed for NTD incidences [10,14], however, few modeling studies have taken into account the spatial autocorrelation of the NTD incidences. Paciorek [18] and Nuckols [19] concluded that there are often markedly spatial autocorrelation in prediction residual in the regression models for public health risk assessment due to factors not captured in the models. The neglect of such residual spatial autocorrelation may result in biased or under-performed model in health risk assessment.

To address the problems above, we developed a spatial model to predict NTD incidences based on environmental variables. A Poisson distribution was used to simulate the incidence of NTD in counts [20]. The generalized additive model (GAM) [21] was used to model the potential non-linear relationships between environmental covariates and the local means of NTD incidences [9]. The residual from GAM is second-order stationary [22] and was modeled through co-kriging with regional residuals at sampling locations nearby. In addition to the model development, we compared our approach with three other methods including universal kriging, generalized linear regression, GAM.

## Methods

### Study domain

**Figure 1:**
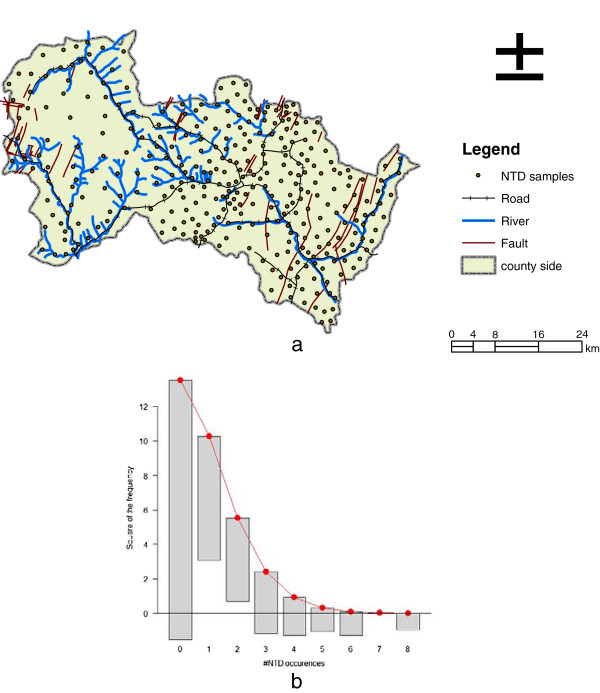
Study region (a), histograms, density plots (b) fitted of expected NTD occurrences during 2002–2005.

Heshun county (Figure 1-a) is located at the Tai Hang mountain area of the Shanxi province, China and consists of 326 administrative villages with a total area of 2,250 km^2^. Most population in this county were farmers who seldom changed their living environment. There was no large-scale human immigration in the region’s history. Remarkably, most kinds of birth defects designated by the World Health Organization have been found in Heshun, and among them the NTD were the predominant birth defects [16]. The inherited and congenital causes explained only a small fraction of the incidences of NTD and they were not distinctly different among the regional populations [16].

### Measured cases of NTD

Annual incidence of NTD from 1998 to 2005 was recorded for each of the 326 sampling villages within Heshun County. Figure 1-a shows the location of each village in the study region. We used the Poisson distribution to simulate the incidences of NTD [20]. Figure 1-b presents the total Poisson distribution fit across the villages (μ = 0.5736, σ^2^ = 1.2914, overexposure = 2.251 with p-value < 0.1).

### Spatial data and covariates

Literature has identified the following factors related to the NTD incidences: deficiency of folic acid and vitamin B_12_[2], chemical elements in soil [11,12], exposure to pesticide and disinfectants [7,8], exposure to nitrate in drinking water [23,24], and geophysical condition [9]. As an agricultural population, farmers in Heshun County frequently used fertilizers, which might end up in soil or water bodies and subsequently increase nitrate levels in drinking water. Other environmental factors such as faults, watershed, rivers, and road might also be associated with pollution in food, drinking water, and air. Accordingly we made an initial selection of the influential factors relevant to NTD as the pool of regressors for further screening.

#### Physical factors

We collected two area-type variables (soil types and lithodological classes) and the locations of four line-type geographical features (i.e. faults, watersheds, rivers, and major roads). We calculated the proportion of area for specific soil and lithodological types within different buffer radius and estimated the shortest distance from the centroid of each village to each fault, watershed, river, and major road. We classified soils into nine types, including cinnamon soil, calcareous cinnamon soil, middle cinnamon soil, neutral lithosol, neutral regosol, cinnamon soil, calcareous regosol, calcareous lithosol, and fluvo-aquic soil. We classified lithodological characters into six classes, including C: coal, fireclay, iron, carboniferous, potassium-bearing rock, silicon rock, sulfur, aluminum; P: Permian rocks, ferromanganese, violet sand earthenware clay; T: middle Triassic rocks; O: limestone, dolomite, middle Ordovician rocks; A: garnet; Q: clay cement grout with mixture of limestone and dolomite.

#### Other influential factors

For each village, we obtained its spatial location (the centroid projected coordinates) as well as other parameters including the total population, the number of doctors in service, gross domestic product (GDP), average annual fruit and vegetable production (kilogram), and the total amount of fertilizer uses (kilogram) per year. GDP reflects socioeconomic status of the population; higher GDP likely indicates a better living condition and foods richer in folic acid, vitamins and other nutrients. The number of doctors reflects local health services and the accessibility of hospital resources, which may influence education on disease prevention and prenatal care (e.g. early identification and abortion of fetus with birth defects).

### Modeling approach

#### Optimal buffering analysis to extract qualitative covariates

The optimal decaying buffering analysis [25] was used to identify the optimal buffering distance for the proportion of area of each type of soils and lithodological classes. We developed a python script to extract the area proportion within a certain buffer distance in ArcGIS 10.0 (ESRI, Redlands, CA). R Statistics 2.14 (R Development Core Team, Vienna, Austria) was used to calculate the Pearson correlation between the target variable (NTD incidences) and the area proportion of each type of soils and lithodological classes across a series of buffer distances (from 20 m to 3 km). A variable was dropped for further analysis if the absolute value of correlation coefficient was less than 0.2 or with p > 0.1 (statistical insignificance).

#### Simulation of Poisson distribution for NTD incidences

Using the Poisson distribution, we simulated the counts of NTD incidences based on the following probability function:

(1) $$f\left(k;\lambda \right)=\;\frac{\lambda^{k}e^{-\lambda }}{k!}$$

*λ* is the expected value of NTD incidences during the entire study period (8 years from 1998 to 2005), and *k* is the assumed counts and *f*(…) corresponds to the probability of *k* NTD incidences during the same period.

#### GAM for an initial estimation of local expected incidences

The general equation of predictive value of NTD incidences:

(2) Λ=μ+e

where *Λ*  =  [*λ*_*i*_](*i*  =  1, …, *n*) is the vector of the target variable (the expected count of NTD incidences) at *n* spatial sampling locations, *μ*  =  [*μ*_*i*_] is the vector of local means at the locations, determined by spatially-resolving covariates, *X =* [*x*_*ij*_] (*i* = 1,…, *n*; *j* = 1,…, *m*; *m* is the number of the covariates), *e*  =  [*ε*_*is*_] is the vector of spatial residual, determined by spatial and regional residuals of samples belonging to the neighborhood of the sampled locations where NTD occurs or not, *e* ∼ *N*(0,  *V* (*θ*)) and are assumed to spatially correlative (*V* (*θ*) representing the resulting covariance matrix determining by spatial autocorrelation coefficients, *θ*).

We used the GAM package in R statistical software (R version 2.14) to estimate local means (*μ* in equation [2]) of NTD incidences. The following is the equation of GAM:

(3) $$\begin{array}{c} g\left(\mu_{i}\right)=\;\mu_{o}\;+\;\sum_{j=1}^{r}f_{j}\left(x_{u}^{j},df\right)\\ \;+\sum_{k=r+1}^{m}\beta_{k}x_{u}^{k} \end{array}$$

where *μ*_0_ is the model intercept, *x*_*u*_^*j*^ or *x*_*u*_^*k*^ ∈  *X* is local covariate such as the proportion of areas for each soil and lithodological type in a certain buffer radius, the shortest distance to fault or river, fertilizer uses, and the number of doctors, *f*_*j*_(…) is the smooth function used to construct the non-linear relationship between *x*_*u*_^*j*^ and g(*μ*_*i*_), *df* is degree of freedom that controls the smooth degree of the curve fit, β_k_ is the linear parameters used to construct the linear relationship between *x*_*u*_^*k*^ and g(*μ*_*i*_), *m* is the total number of covariates. Here we assumed that there are non-linear relationships between *r* covariates and g(*μ*_*i*_). For Poisson distribution, we used log link function for *Λ* in [1]: *μ*  =  [*λ*_*i*_]  =  [*μ*_*i*_]  =  [exp(*g*(*μ*_*i*_))] or g(*μ*_*i*_) = log(*μ*_*i*_) (for Poisson distribution, *λ*_*i*_  =  *μ*_*i*_ representing the expected count of NTD incidences).

Three steps were used to select the covariates in GAM. First, to avoid multi-collinearity, we used variance inflation factor (VIF) to divide the covariates into two parts: weakly correlated covariates (VIF < 5) and the independent groups of highly correlative ones (VIF > =5) (correlation coefficients were used to divide the highly correlative covariate into different groups) [26]. Then, each covariate was in turn selected from each group of the highly correlative covariates (only one covariate selected from each highly correlative group each time) and combined with the weakly correlative covariates to construct a model’s combination of covariates. R^2^ was used to backward-select each combination of covariates with statistical significance (p-values <0.1): the covariates that did not reach statistical significance were removed until R^2^ remained the same, improved, or decreased the least when all possible combinations of remaining covariates were considered. Finally, the covariates having the maximum R^2^ were selected as the optimal set from the groups of covariates.

#### Co-kriging spatial residuals to minimize error variance

Assuming a stable space domain after the modeling of local means using GAM [22], we used regional residual (representing regional variability of the expected incidence under the entire study domain due to spatial variability from geospatial factors not captured in the model) to co-krige the residual from GAM.

(4) $$\varepsilon_{\mathrm{is}}\;=\;\sum_{j=1}^{n_{i}}\lambda_{j}^{s}\varepsilon_{s}\left(j\;\in \;N\left(i\right)\right)\;+\;\lambda_{j}^{r}\varepsilon_{s}\left(j\;\in \;N\left(i\right)\right)$$

Where *ε*  =  *ε*_*is*_ ∼ *N*(0, *V*(*θ*)), *θ* is the vector of variogram parameters, *j ∈ N(i)* is the set of neighborhood samples around *u* (*n*_*i*_ is the number of neighborhood samples), *ε*_*s*_(*j*) is the estimate of spatial residuals at *j*, and is derived by subtracting the GAM-predicted expected NTD incidences from the measured or observed concentration at each sampling site, *ε*_*r*_(*j*) is the estimate of regional residual or the total variation of the measured values at a regional or background scale under the study domain, derived by subtracting the regional mean (average of the actual NTD incidences at all sites) from the measured values (NTD incidences) at each sampling site. *λ*_*j*_^*s*^ and *λ*_*j*_^*r*^ are the optimal weights estimated using maximum likelihood based on the spatial coefficients (sill and range) of *θ*[27,28].

The residuals are influenced by both local variation of the target variable (expected NTD incidences) and regional variation or other unaccounted effects at nearby locations that is referred as regional residual in our model. According to the optimal principle of unbiased estimation and minimal error variance of co-kriging, if the variogram of spatial and regional residuals is precisely captured, error variance of spatial residual will decrease substantially and in turn *R*^2^ will increase [29]. Variogram represents the degree of spatial dependence of a spatial random field or stochastic process and reflects a feature’s variation along a certain spatial distance in a spatial field [27]. A longer range and a smaller sill indicate a less heterogeneous or a slower changing spatial surface. Thereby, we co-kriged the spatial residual with regional residual at surrounding sampling locations to improve the prediction.

We used the theoretical variogram to fit the experimental variogram of spatial and regional residuals and the cross covariance between them. We derived the cross covariance or the change of the variance between the spatial residual and the regional residual from the variogram. We examined the semi-variogram cloud, tested different lag sizes and numbers of lags, and different variogram models to find the best reasonable fit by cross validation using ArcGIS (Version 10.0)’s Geostatistical Analyst [28].

### Model comparison

We compared our modeling approach (GAM plus cokriging of spatial residuals) to three other methods, i.e. universal kriging, generalized linear regression (GLM), and GAM only. Universal kriging estimates local means using coordinates and the residuals, but it has no structure to take into account local spatial covariates and regional variability. GLM assumes a linearly additive relationship between expected means and all spatial covariates. GAM incorporates both linear and nonlinear relationships but GAM itself does not account for spatial autocorrelation of the residuals. The GAM plus cokriging model incorporates both variability of local means estimated with GAM and the large-scale regional variability useful for decreasing the bias of the prediction due to factors not accounted in the model [18].

### Cross validation

For model evaluation we used leave-one-out cross-validation (LOOCV). LOOCV involves using a single observation from the original sample as the validation data, and the remaining observations as the training data; this is repeated such that each observation in the sample is used once as the validation data. We used three continuous error measures (*R*^2^, inter-quartile range (IQR) of prediction error, the square root of the mean of the squared prediction errors (RMSPE)) and box plot of the precision error to compare the performance of the models. Prediction error was defined as the difference between the observed and predicted values at each cross-validation measurement location. Box plot was used to visually examine mean, IQR, outliers and the 95% confidence interval of prediction errors. A good model usually has a bigger *R*^2^, a smaller IQR and RMSPE, a mean and IQR close to 0, and narrower confidence intervals of the error in the box plot.

### Inference and prediction of the NTD incidences

With the expected NTD incidence estimated, we used the Poisson probability distribution (Equation [1]) to infer and predict the risk of NTD incidences. With the following equation, we can predict the probability of at least one NTD case during the same 8-year period for each village:

(5) $$p_{j}\;\left(k\geq 1\right)\;=\;1-f\left(0;\lambda \right)$$

Also, we can infer the odds for NTD incidences:

(6) $$odd_{j}\;=\;\frac{p_{j}\left(k\geq 1\right)}{p_{j}\left(k=0\right)}\;=\;\frac{1}{f\left(0;\lambda \right)}\;-1$$

Given the number of total births during the study period, we can estimate the incidences of NTD and identify the spatial variability and potential hot spots.

### Uncertainty analysis

Using the mgcv package for GAM in R, we obtained the 95% upper and lower pointwise confidence limits around the GAM estimate for each regressor. The uncertainty of each covariate was evaluated based on these confidence limits. Further, we tested the sensitivity of the smooth functions of regressors by adjusting the degrees of freedom (5–10). In addition, we examined the uncertainty of the model using different variograms (spherical, circular, exponent, Gaussian and stable).

## Results

### Determinants

We selected one out of nine types of soil (calcareous and lithosol soil) and two out of six lithodological classes (brick clay and Trias Liujiagou group) as the predictive covariate with the corresponding optimal buffer distance resulting in the highest correlation with the target variable. Additional file 1: Figure S1 presents the curve of Pearson correlation between the soil type (a) or the lithodological types (b and c) with the NTD incidences. Calcareous lithosol soil and brick clay lithodological class each positively correlated with the NTD incidences (optimal buffering distance: 1.5 km and 2.5 km; correlation: 0.30 and 0.21) but the Trias Liujiagou group lithodological class negatively correlated with the NTD incidences (optimal buffering distance: 1.5 km; correlation: -0.23). Other covariates were removed from the model due to their weak correlation (<0.1) with the target variable.

From eleven covariates (one soil type, two lithodological classes, the projected x/y coordinates, distance to watershed, GDP, number of doctors, average yearly fertilizer uses, shortest distances to rivers, faults and roads), five covariates, namely x/y coordinates, fertilizer uses, shortest distance to faults, shortest distances to rivers, lithodological type of Trias Liujiagou group (T*) were selected as the final predictive as determinants. Table 1 lists the variance explained and statistical significance at the *α* = 0.05 level for each selected covariate. Additional file 1: Figure S2 shows the non-linear relationship between local covariates and the expected NTD incidences modeled by GAM. In this figure, fertilizer uses (Additional file 1: Figure S2-a) and lithodological type (Q*) (Additional file 1: Figure S2-f) had a non-linear positive correlation with expected NTD incidence (abbreviated as #NTD) (a curve of incremental trend with small fluctuations). While the shortest distance to the faults was non-linearly negatively associated with #NTD, the shortest distance to rivers was non-linearly positively associated with #NTD (a curve of gradually incremental trend with more fluctuates, Additional file 1: Figure S2-c): within a 2 km distance, living farther to the rivers increased the risk of NTD, but no clear trend was observed after 2 km. On the other hand, lithodological type (T*) and lithosol soil respectively represent the markedly monotone decreasing and increasing linear relationship with #NTD. We found that GDP, fruit and vegetable production, number of doctors, and the total number of population were insignificant predictors thus were not used in the model.

**Table 1:** Spatial covariates selected for GAM (after removing colilinearity)

**Spatial Covariates selected**	**Buffer distance (m)**	**R**	**V.P**	**F**	**p-value**
Geographic location	-	-	19.03	3.828	7.53e-7*
Fertilizer used	-	0.40	25.64	14.74	<2e-16*
Shortest distance to faults	-	−0.14	4.96	2.499	0.01*
Shortest distance to rivers	-	−0.22	7.52	4.149	5.12e-5*
Lithodological type of Trias Liujiagou Group (T*)	1500	−0.23	1.04	0.03	0.0328*
Calcareous lithodological soil	2500	0.21	-	19.14	1.64e-5*
Brick clay lithodological type(Q*)	1500	0.30	-	16.19	3.78e-9*

*V.P*: the percent of variance explained by the factor variable (%) in GAM; *R*: Pearson correlation; *F*: statistics of ANOVA test; p-value: statistical significance.

### Spatial correlation

Variogram models showed spatial autocorrelation of local and regional residuals (Additional file 1: Table S1 and Figure S3 for optimal variogram results). As shown in Additional file 1: Table S1, the range of local residual is 2425.41 m, much lower than that of regional residual (13836.0 m). Local residual also had a smaller sill (=nugget + partial sill, 0.65 m) than regional residual (1.47 m). The variogram curves (Additional file 1: Figure S3) clearly demonstrate that regional residual had a wider influence on the target variables (#NTD, longer range and bigger sills). Cross covariance between spatial and regional residuals was moderate between local and regional residuals.

### Comparison of models

**Figure 2:**
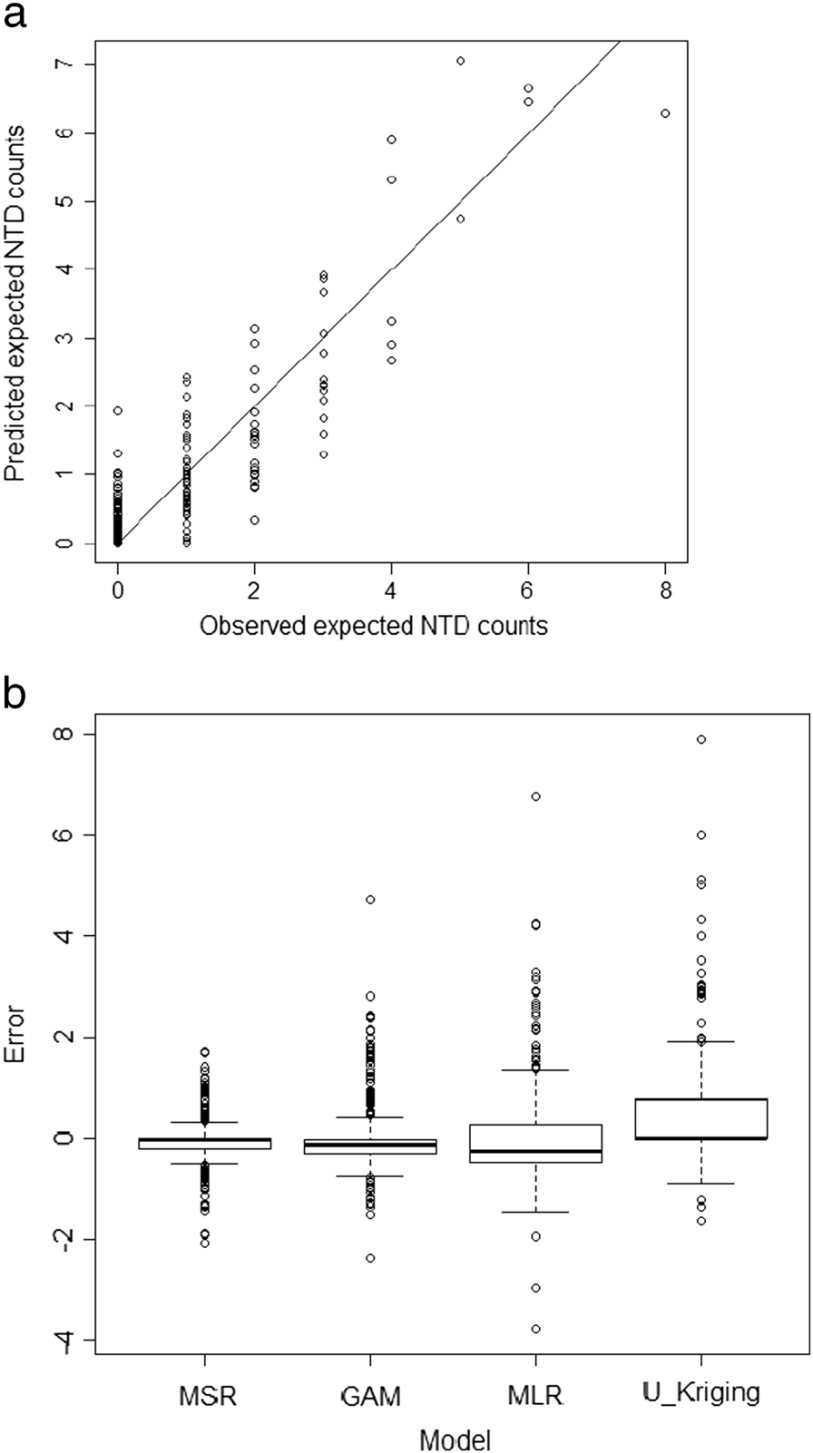
**Plot of observed NTD counts vs. expected NTD counts predicted by our method (a, *R***^**2**^ **= 0.804) and box plots (b) of precision errors for four models in NTD expected occurrences (error bars indicate 95% confidence intervals; circles indicate outliers).**

Our spatial residual model had the highest cross validation *R*^2^ (CV *R*^2^, 0.80, Figure 2-a), close-to-zero IQR (−0.045), and the smallest RMSPE (0.50) among the four models (Table 2). Box plot of prediction errors had narrower 95% confidence intervals, closer-to-zero median, and fewer outliers for our model (Figure 2-b). Three measures of predictive residuals (CV *R*^2^, IQR and RMSPE, Table 2) and box plots (Figure 2) illustrate that our model achieved the best prediction performance among the four models. Co-kriging residual with regional residual accounted for 22.2% of the variability of NTD incidences and improved R^2^ up to 38%, a substantial increase in prediction over GAM without considering spatial autocorrelation of residuals.

**Table 2:** Comparison of predictive errors in four models by leave-one-out cross validation

**Models**	***CV R***^**2**^	***M***	**RMSPE**
Generalized linear regression	0.234	−0.25	0.993
Universal kriging	0.164	0.414	1.17
GAM	0.582	−0.135	0.734
Our model of spatial residual	0.804	−0.045	0.502

*CV R*^2^-cross validation *R*^2^; *M*: Median (IQR) of prediction error.

*RMSPE*: the squared root of the mean of the squared prediction errors.

### Risk mapping

**Figure 3:**
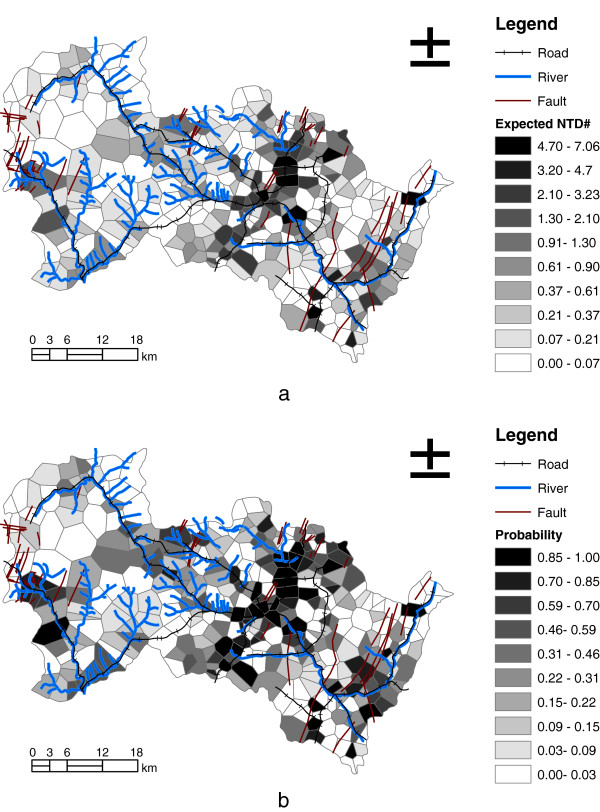
Prediction of the expected NTD occurrences inferred (a) and probability prediction of one NTD at least one birth defects (b).

**Figure 4:**
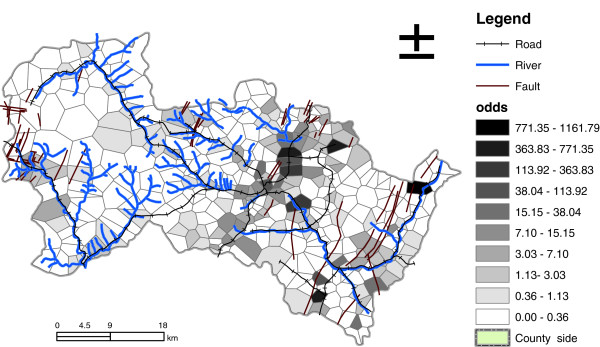
Odds ratio map of at least one birth defects inferred.

Figure 3-a shows the expected incidences of NTD for each village during the 8 years using our residual spatial model and Figure 3-b represents the probability risk of at least one NTD during this period (equation [5]). Figure 4 shows the odds of NTD incidence to non-NTD incidence according to equation [6]. According to the odds map, several villages of hot spots with high risk of #NTD (odds > 500) were identified, including Qing Cheng, Bo Li, Xu Cun and Niu Chuan.

### Uncertainty analysis

Uncertainty analysis showed that our model had a stable prediction performance. Additional file 1: Figure S2 shows the 95% upper and lower pointwise confidence limits around the GAM estimate for each regressor. Further, by adjusting the degrees of freedom for parameters and using different variograms, the median and mean of R^2^ are respectively 0.81 and 0.79 with the 95% confidence intervals of [0.77, 0.81].

## Discussion

We developed a GAM plus cokriging model to estimate the expected incidences of NTD (*R*^2^: 0.80) for Heshun County, Shanxi, China. This study adds values to the literature of environmental risk assessment of birth defects in two aspects. First, to our knowledge, it is one of the first studies of environmental health risk of birth defects that accounts for geographical variability of multiple environmental covariates in combination with spatial autocorrelation of the residuals. Second, by incorporating both non-linear relationships and residual spatial autocorrelation, our GAM plus cokriging model was proved to be an effective modeling approach compared to the other three commonly used methods.

We found that fertilizer uses, residual spatial autocorrelation, and the projected coordinates were important predictors of the NTD incidences, respectively accounting for 25.64%, 22.20%, and 19.03% of the variance. Our results of the fertilizer uses agree with the other environmental epidemiological studies of birth defects [23,30,31], which showed that fertilizer uses may result in increased nitrate exposure from drinking water associated with increased NTD risk. Winchester et al. [32] indicated that there may be synergistic effect of nitrate and pesticides: the combination of pesticides and fertilizer (nitrate) caused increased incidence of fetal abnormalities than just pesticides alone. In this study, no pesticide use data were available to test the combinational effect of fertilizer uses with pesticide uses on the increase of NTD incidences but such a synergistic effect is possible. We also found significant spatial autocorrelation of NTD incidences, consistent with the previous studies [14,16,33]. The coordinates [34] and residual spatial autocorrelation [18] represent spatial variability of environmental risk factors not included in the GAM. Although improving model performance by incorporating spatial autocorrelation of residuals and projected coordinates may mask the importance of certain prediction variables not accounted for in the models, incorporating such spatial information is a practical approach for improving prediction of NTD incidences in epidemiologic studies when important prediction variables is unavailable [18,35]. In addition to ArcGIS’s Geostatistical Analyst (based on the ad hoc fitting methods), we used the geoR package (based on maximum likelihood) in *R* Version 14.0 to model the spatial autocorrelation. The results were similar between the ArcGIS and the geoR estimates.

Additionally, we further observed that higher NTD incidences were associated with living closer to the faults and living in areas with more Q* rock and calcareous lithosol soil, while lower NTD risk was linked to areas with more T* rock. Our findings of the influence of local covariates and the spatial autocorrelation agreed with the work of Wang et al. [16], which also showed the influence of lithozone, soil and faults on the incidences of NTD. Distance to river (within 2 km) was positively associated with the risk of NTD, likely because people living far away from a river may not have frequent access to fresh river water thus they may use stored water (more likely to be contaminated by microorganisms) at home and use water less often for hygiene purposes. The influential zone of a river is likely within 2 km and beyond which the influence is minimal. Our study also found significant association of lithodological type (T*) and lithosol soil with NTD. Lithosol soil may be associated with heavy metal pollution, while Lithodological type (T*) may present beneficial geological environment for decreased NTD incidences.

Although statistical insignificant in our model, fruit and vegetable production (proxies of folic acid deficiency) was found to partly contribute to the distribution of NTD incidences in Heshun County by geographical detector [9]. Liao et al. [15] found that the prevalence of NTD decreased with increased distance from coal plants. Unfortunately, we did not have data on coal plants in this study, but we identified other important predictors that yielded a highly predictive model.

We generated the risk map and identified the hotspot villages with high risk of NTD incidence, which will help prioritize the resources needed for government intervention to reduce the risk of NTD. For the hot spots like Qing Cheng, Bo Li, Xu Cun and Niu Chuan (Figure 4), the local government may need to invest more resources to combat the high risk of NTD. Previous studies showed that the advocating of folic acid supplements has decreased the NTD incidences in high risk communities [2,36]. The results of our study showed that decrease in fertilizer uses might be helpful for lowering the NTD risk [23,24] in these hot spots. Furthermore, based on our study [9], if the local residents can, have access to cleaner water resources [32], access to safer/cleaner or more productive soil [11,24,32], or relocate farther from the faults [9], it is possible to decrease the NTD incidence risk. But some factors may be the surrogates and more survey is required for the deterministic influence of these factors, requiring mechanic knowledge, i.e. the in-depth mechanism for high NTD risk. The relocation may be the least desirable choice due to the high cost and feasibility.

This study has several major limitations. First, we did not have data on the number of births for the period of data gathering thus we did not calculate the rate of NTD incidences. But the Poisson probability model is suitable for dealing with the probability assessment of count events and the output of odd ratios of birth defects to non birth defects reasonably reflects spatial variability of NTD risk (Figure 4 and Equation 6). Second, due to missing NTD data for certain villages at the beginning couple of years and the lack of temporal trend data for the influential covariates, we averaged the expected NTD incidences over the eight year period (1998–2005) and considered no temporal trend in our model. Third, our method might have a problem of over fitting. However, this problem was mitigated to the least in three aspects: we used leaving-one-out cross validation to validate the model that has been proved to perform well for continuous error measurement [37]; the degree of freedom in GAM was set to be less than 10 for most covariates, thus avoiding the over-fitting problem of the GAM. Although improvement in prediction of NTD# by incorporating spatial autocorrelation of residuals may mask significance of certain physical determinants, such an incorporation of residuals is a practical approach for substantial improvement in prediction of NTD when other covariates were not so predictive and the improvement is very significant for monitoring and preparedness of birth defects.

## Conclusion

This study developed a residual spatial model that coupled GAM and co-kriging to assess spatial variability of the expected incidence of NTD and its risk in Heshun County, Shanxi Province, China. Our method examined the influences of local environmental covariates, including shortest distances to faults and rivers, soil, rock, fertilizer uses and spatial location upon the variability of NTD incidences. Our method used GAM to establish the linear/non-linear relationship between the covariates and the NTD risk and used co-kriging to incorporate spatial autocorrelation of residuals from GAM. Compared with the other three methods, our method achieved the better effect with its LOOCV R square of 0.80. Our study has significant implication for the epidemiological studies of the influence of environmental factors on birth defects.

## Abbreviations

NTD: Neural tube defects; GAM: Generalized additive model; GLM: Generalized linear regression; CV: Cross validation; LOOCV: Leave-one-out cross-validation; IQR: Inter-quartile range; RMSPE: Square root of the mean of the squared prediction errors.

## Competing interests

The authors declare they have no competing interests.

## Authors’ contributions

LL ideated this paper’s content, developed the model, analyzed the data and jointly drafted and revised the manuscript. JW1 (Jinfeng Wang) provided the background knowledge and critical data support, gave constructive modeling suggestions and jointly revised this paper. JW2 (Jun Wu) helped improve the modeling, provided relevant background knowledge and perfect interpretation of the results, and made a considerable contribution to the paper’s revision. All authors read and approved the final manuscript.

## Pre-publication history

The pre-publication history for this paper can be accessed here:

http://www.biomedcentral.com/1471-2458/12/951/prepub

## Supplementary Material

Additional file 1 **Supplemental Materials. Table S1.** Optimal fitted variogram models of local and regional residuals by cross validation. **Figure S1**. Correlation of the soil (a) and lithodological types (b, c) along the decaying buffer distances. **S2**. Non-linear/linear relationship between local covariates and the expected NTD incidences modeled by GAM. **S3**. Variograms of local and regional residuals and cross covariance. (DOCX 160 kb) Click here for file
