# Supplementary material for: A spatial model to predict the incidence of neural tube defects
Source: BMC Public Health. 2012 Nov 7;12:951. doi: 10.1186/1471-2458-12-951 (PMC3556316; doi:10.1186/1471-2458-12-951)
Supplement: Additional file 1 — Supplemental Materials. Table S1. Optimal fitted variogram models of local and regional residuals by cross validation. Figure S1. Correlation of the soil (a) and lithodological types (b, c) along the decaying buffer distances. S2. Non-linear/linear relationship between local covariates and the expected NTD incidences modeled by GAM. S3. Variograms of local and regional residuals and cross covariance. (DOCX 160 kb) [file 1471-2458-12-951-S1.docx]

**Supplemental Materials**

for the paper titled A Spatial Model to Predict the Incidence of Neural Tube Defects

Table S1. Optimal fitted variogram models of local and regional residuals by cross validation

| Model | L_R | R_R | C_V |
| --- | --- | --- | --- |
| Model type | Spherical | Stable | Spherical |
| Parameter | - | 0.697 | - |
| Major range | 2425.41 | 13836 | 2543.79 |
| Nugget | 0.606 | 1.167 | 0 |
| Partial sill | 0.043 | 0.303 | 1.261 |

Note: L_R—local residual; R_R—regional residual;

C_V-- cross variogram between local and global residuals


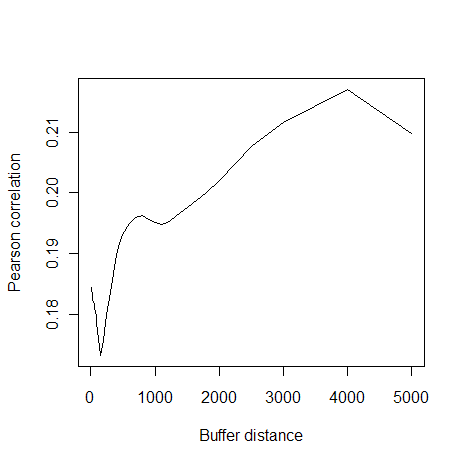


a). Calcareous lithosol


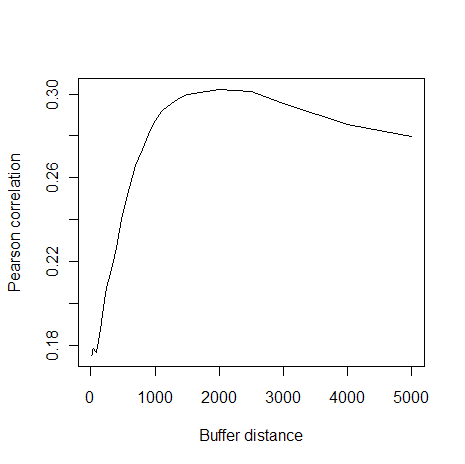

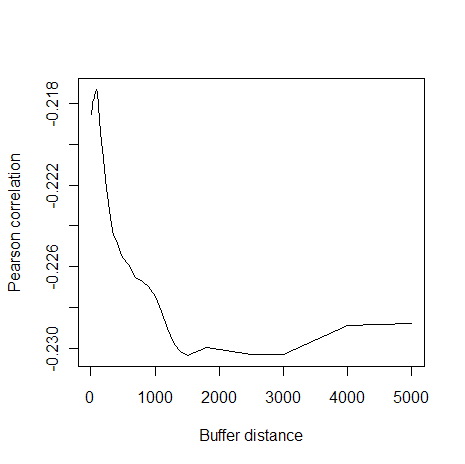


b). Brick clay lithodological type(Q*) c). Trias Liujiagou Group lithodological type (T*)

Figure S1. Correlation of the soil (a) and lithodological types (b, c) along the decaying buffer distances


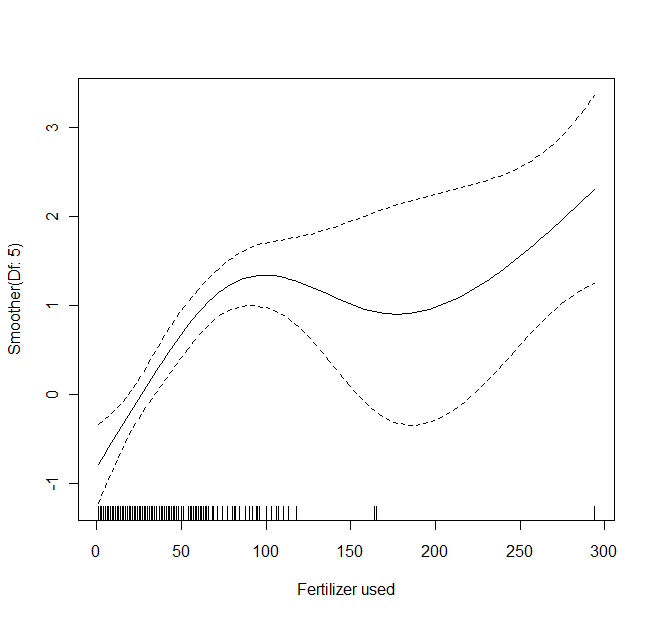

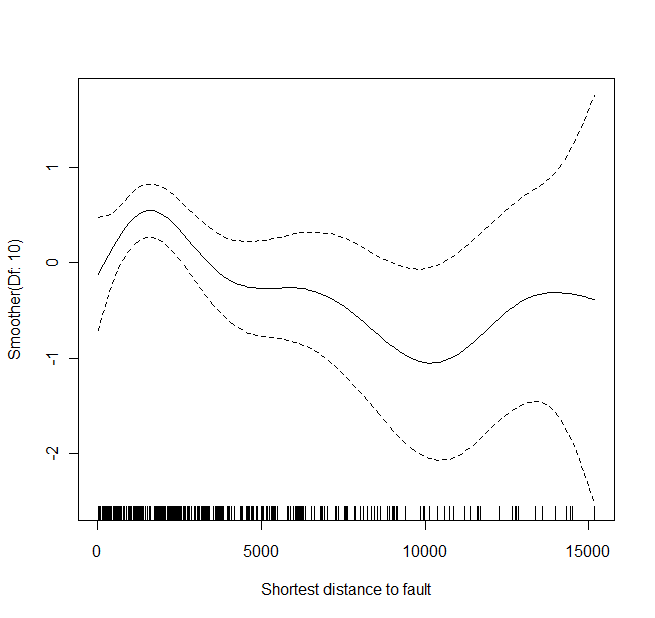


a b


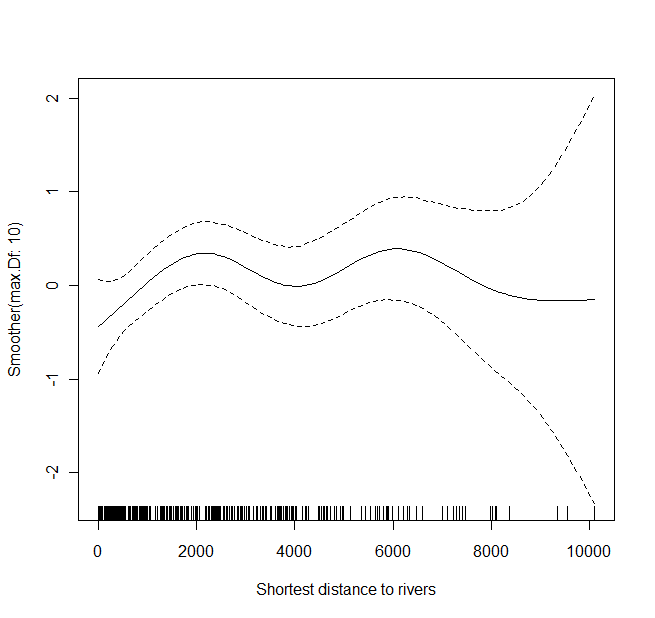

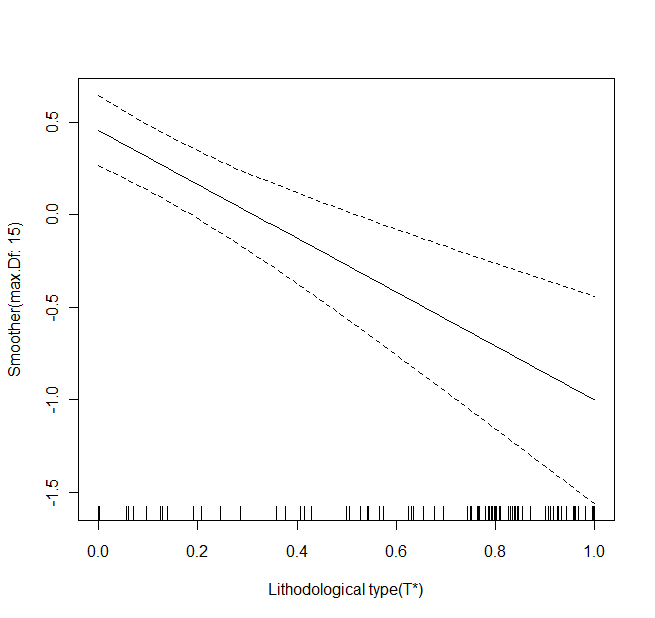


c d


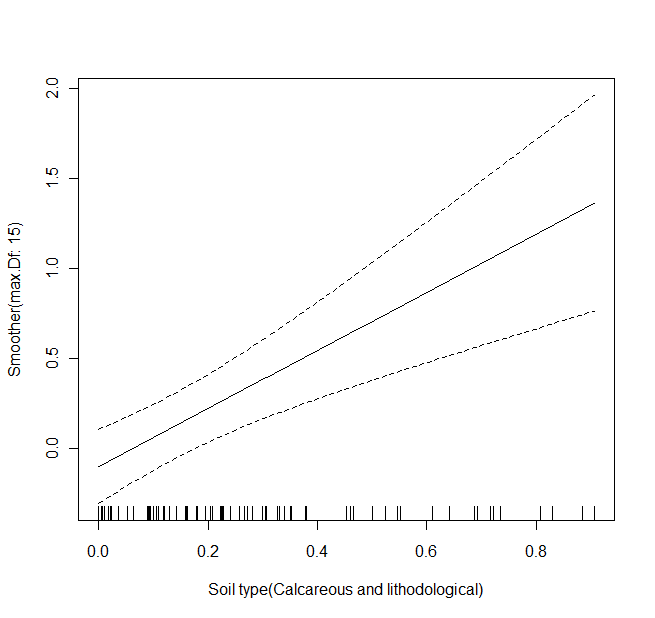

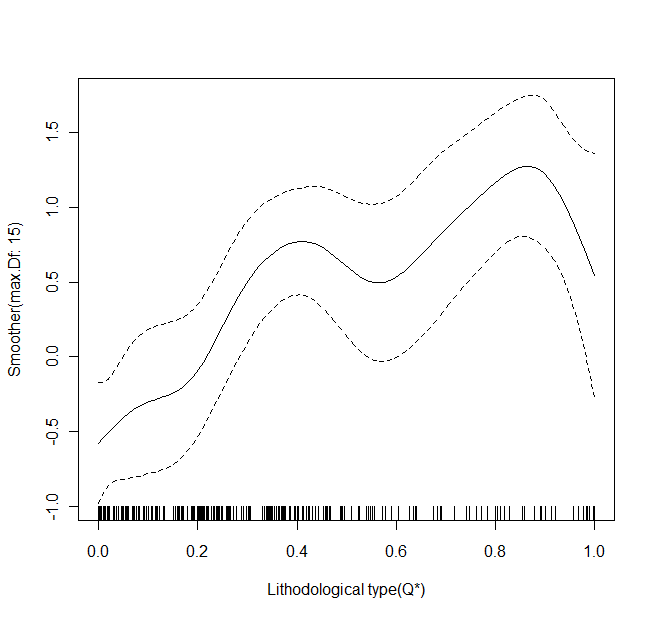


e f

Figure S2. Non-linear/linear relationship between local covariates and the expected NTD incidences modeled by GAM


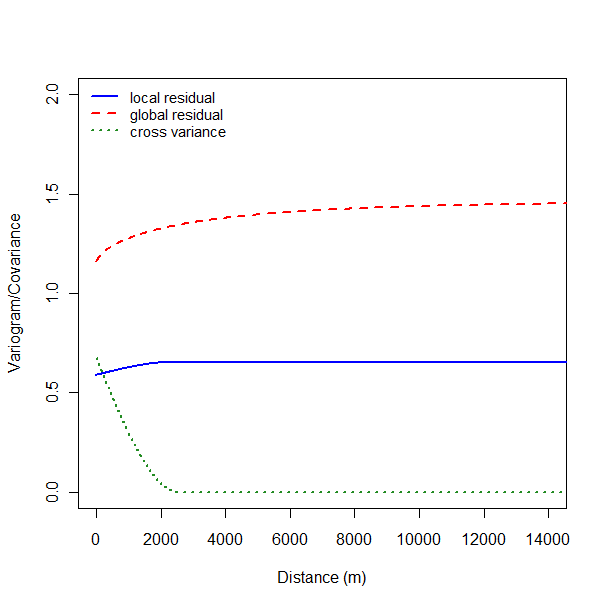


Figure S3 Variograms of local and regional residuals and cross covariance
